# Discovery of a Novel Class of Norovirus Inhibitors with High Barrier of Resistance

**DOI:** 10.3390/ph14101006

**Published:** 2021-09-30

**Authors:** Jana Van Dycke, Michela Puxeddu, Giuseppe La Regina, Eloise Mastrangelo, Delia Tarantino, Jasper Rymenants, Jessica Sebastiani, Marianna Nalli, Jelle Matthijnssens, Johan Neyts, Romano Silvestri, Joana Rocha-Pereira

**Affiliations:** 1 Laboratory of Virology & Chemotherapy, Department of Microbiology, Immunology & Transplantation, Rega Institute for Medical Research, KU Leuven—University of Leuven, 3000 Leuven, Belgium; jana.vandycke@kuleuven.be (J.V.D.); jasper.rymenants@kuleuven.be (J.R.); johan.neyts@kuleuven.be (J.N.); 2 Laboratory Affiliated with the Institute Pasteur Italy—Cenci Bolognetti Foundation, Department of Drug Chemistry and Technologies, Sapienza University of Rome, Piazzale Aldo Moro 5, 00185 Rome, Italy; michela.puxeddu@uniroma1.it (M.P.); giuseppe.laregina@uniroma1.it (G.L.R.); jessica.sebastiani@uniroma1.it (J.S.); marianna.nalli@uniroma1.it (M.N.); romano.silvestri@uniroma1.it (R.S.); 3 CNR—Biophysics Institute, Università degli Studi di Milano, 20122 Milano, Italy; eloise.mastrangelo@unimi.it (E.M.); delia.tarantino@unimi.it (D.T.); 4 Laboratory of Clinical and Epidemiological Virology, Department of Microbiology, Immunology & Transplantation, Rega Institute for Medical Research, KU Leuven—University of Leuven, 3000 Leuven, Belgium; jelle.matthijnssens@kuleuven.be

**Keywords:** *Caliciviridae*, norovirus, in vitro, small molecule

## Abstract

Human noroviruses (HuNoVs) are the most common cause of viral gastroenteritis resulting in ~219,000 deaths annually and a societal cost of ~USD60 billion. There are no antivirals or vaccines available to treat and/or prevent HuNoV. In this study, we performed a large-scale phenotypical antiviral screening using the mouse norovirus (MNV), which included ~1000 drug-like small molecules from the Drug Design and Synthesis Centre (Sapienza University, Rome). Compound 3-((3,5-dimethylphenyl)sulfonyl)-5-chloroindole-N-(phenylmethanol-4-yl)-2.carboxamide (compound **1**) was identified as an inhibitor of MNV replication with an EC_50_ of 0.5 ± 0.1 µM. A series of 10 analogs were synthesized of which compound **6** showed an improved potency/selectivity (EC_50_ 0.2 ± 0.1 µM) against MNV; good activity was also observed against the HuNoV GI replicon (EC_50_ 1.2 ± 0.6 µM). Time-of-drug-addition studies revealed that analog **6** acts at a time point that coincides with the onset of viral RNA replication. After six months of selective pressure, two compound **6**^res^ variants were independently selected, both harboring one mutation in VPg and three mutations in the RdRp. After reverse engineering S131T and Y154F as single mutations into the MNV backbone, we did not find a markedly compound **6**^res^ phenotype. In this study, we present a class of novel norovirus inhibitors with a high barrier to resistance and in vitro antiviral activity.

## 1. Introduction

Human norovirus (HuNoV) is a (+)ssRNA virus, that belongs to the *Caliciviridae* family which causes gastroenteritis resulting in vomiting and watery non-bloody diarrhea. HuNoV infections occur worldwide and are an important health issue that affects all age groups. After the introduction of two rotavirus vaccines, HuNoV is becoming the most common cause of viral gastroenteritis, resulting annually in 200,000 deaths [1]. Infections occur via the fecal-oral route, by consumption of contaminated food or water and by person-to-person contact. HuNoV is very contagious and resistant in the environment; therefore, large outbreaks occur frequently and most often in health institutions, long-term care facilities and other semi-closed environments [2]. The current therapy consists merely of supportive supplementation with oral rehydration salts (ORS). There are still no antivirals to treat and/or prevent HuNoV infections. An efficacious anti-norovirus drug that could be used as a prophylactic or to treat HuNoV infections is urgently needed.

In the search of new anti-norovirus molecules, we performed a large-scale antiviral drug screening, which included ~1000 drug-like small molecules from the Drug Design and Synthesis Centre (Sapienza University, Rome). Due to the lack of a robust and high-throughput cell culture system for HuNoV, the screen was performed using mouse norovirus (MNV). From the initial screening, we identified 3((3,5 dimethylphenyl)sulfonyl) 5 chloroindole-N (pheylmethanol 4 yl)-2-carboxamide(compound **1**) as an interesting hit compound. Based on the initial results, a test set of 10 analogues, **2–11**, was synthesized to further explore this class of norovirus inhibitors; among them, analog **6** showed an improved potency and selectivity against MNV and was studied in more detail.

## 2. Results

### 2.1. Compound Synthesis

**Scheme 1:**
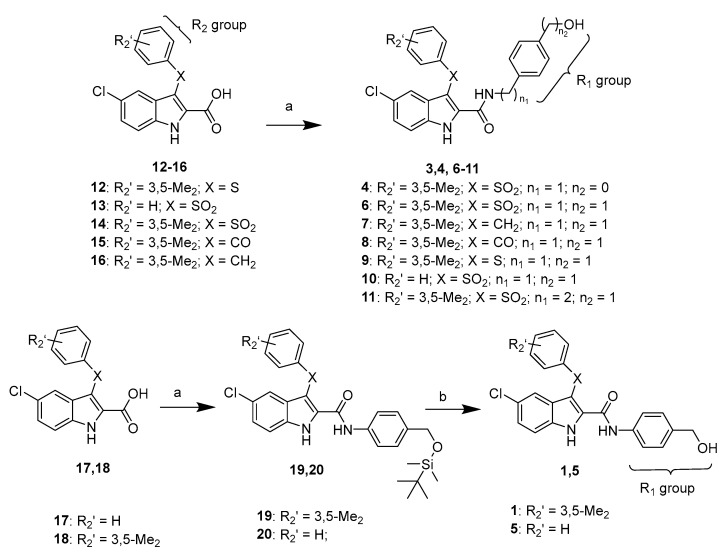
Synthesis of compounds **1** and **4**–**11**. Reagents and reaction conditions: (a) appropriate amine, (benzotriazol-1-yloxy)tripyrrolidinophosphonium hexafluorophosphate, triethylamine, *N*,*N*-dimethylformamide, 40 °C,18 h, Ar (b) tetrabutylammonium fluoride, tetrahydrofuran, 25 °C, 4 h.

Compounds **1** and **5** were obtained by treatment of compounds **19** and **20**, respectively, with tetrabutylammonium fluoride (TBAF) in tetrahydrofuran at 25 °C for 4 h (Scheme 1). Compounds **2** and **3** were prepared as previously reported. Compounds **4**, **6**–**11** and **19**, **20** were obtained by reaction of acids **12–18** with the appropriate amine in the presence of (benzotriazol-1-yloxy)tripyrrolidinophosphonium hexafluorophosphate (PyBOP) and triethylamine in *N*,*N*-dimethylformamide at 40 °C for 18 h under argon stream (Scheme 1).

### 2.2. Inhibition of MNV Replication and the HuNoV GI.1 Replicon

Compounds **1**, **6**, **7** and **9**–**11** inhibited the MNV induced cytophatic effect (CPE) with EC_50_ values ranging from 0.16 µM (compound **6**) to 35.95 µM (compound **11**), and five compounds (**2–5** and **8**) showed EC_50_′s >100 µM. In this assay, the cytotoxic concentrations (CC_50_ values) of compounds **1**, **2** and **6** were >100 µM, and those of compounds **3–5** and **7–11** were in the 3.48 (compound **4**)–88.69 (compound **3**) µM range.

**Figure 1:**
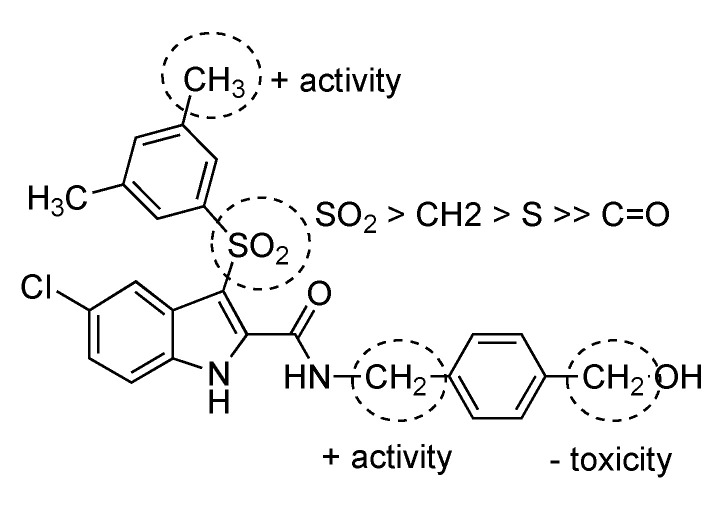
Structure-activity relationships summary.

Replacement of the N-4-(hydroxymethyl)phenyl group at the indole-2-carboxamide nitrogen of compound **1** with the homologue N-4-(hydroxymethyl)benzyl (compound **6**) improved the inhibitory activity against both MNV and HuNoV replicon, meanwhile the CC_50_ value was >100 µM. The full N-4-(hydroxymethyl)benzyl end tail played a key role for the inhibitory activity. In fact, if a compound is deprived of the 4-hydroxymethyl group (compound **2**) [3], of the phenyl ring (compound **3**) [4], or bears the isomeric phenol tails (compounds **4** and **11**) the activity was remarkably less compared to compounds **1** and **6** (phenol derivatives **4** and **11** were also highly cytotoxic). Replacement of the sulfonyl bridging group by a methylene, carbonyl, or sulfur group provided less active or inactive compounds against both strains (compare compound **6** with compounds **7–9**). Compounds **1** and **6** with the 3,5-dimethylsulfonyl group at position 3 of the indole were superior to the corresponding 3-phenylsulfonyl derivatives **5** and **10**. Compound **6** was the most potent inhibitor of both MNV and the HuNoV replicon within the series, with EC_50_ values of 0.16 µM and 1.24 µM, respectively, and CC_50_ >100 µM. Thus, compound **6** was selected for more in-depth in vitro studies (Table 1 and Figure 1).

### 2.3. Compound **6** Inhibits the Replication of MNV and the HuNoV GI.1 Replicon Most Efficiently

**Figure 2:**
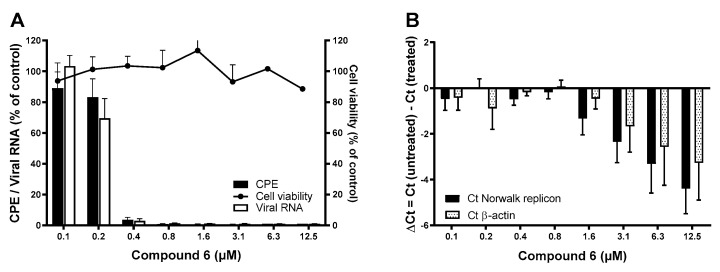
**The antiviral effect of compound 6 against MNV and the HuNoV GI.1 replicon.** (**A**) The antiviral activity of compound **6** was quantified by means of (i) virus-induced CPE reduction assay using a colometric read-out (black bars) and (ii) viral RNA levels using RT-qPCR (empty outlined bars). Results are mean values (±SEM) of 6 independent experiments. Potential adverse effects on the host cells were assessed in parallel and quantified by a colometric assay (black dots). Results are mean values (±SEM) of 2 independent experiments. (**B**) The antiviral activity of compound **6** against the HuNoV GI.1 replicon was quantified by RT-qPCR. Intracellular RNA loads are represented by absolute genome quantification of the HuNoV GI.1 replicon versus β-actin. Results are mean values (±SEM) of 8 independent experiments.

A series of analogs of 3-((3,5-dimethylphenyl)sulfonyl)-5-chloroindole-N-(phenylmethanol-4-yl)-2.carboxamide (compound **1**) were synthesized and tested for anti-norovirus activity. Out of these, compound **6** had improved antiviral activity against MNV (EC_50_ (CPE) of 0.16 ± 0.06 µM, EC_50_ (viral RNA) of 0.21 ± 0.03 µM), with a 50% toxic concentration (CC_50_) >12.50 µM (Figure 2). Moreover, compound **6** remained active against the HuNoV GI.1 replicon with an EC_50_ value of 1.27 ± 0.76 µM and without adverse effects on the host cells (Table 1).

### 2.4. Compound **6** Acts at the Onset of Viral Replication

**Figure 3:**
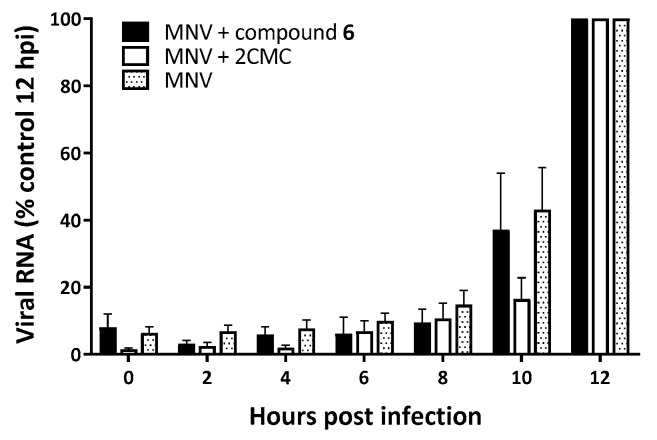
**Time-of-drug-addition (TOA) studies of MNV-infected RAW264.7 cells, either untreated or treated with compound 6 or the nucleoside analog 2CMC.** TOA studies of RAW264.7 cells infected and untreated (dotted bar) and RAW264.7 cells infected and treated with compound 6 (black bars) or 2CMC (empty outlined bars). Results are mean values (±SEM) of 3 independent experiments.

To assess at which moment compound **6** acts on the MNV replication cycle, we performed a time-of-addition (TOA) experiment (Figure 3). First, the kinetics of a single replication cycle was determined in untreated infected cells. Intracellular viral RNA starts to increase at 6–8 h pi, thus marking the beginning of viral genome synthesis. If compound **6** was added later than 6–8 h pi, the antiviral effect was lost. This assay revealed that compound **6** acted at the onset of the viral genome replication, like the nucleoside 2′-*C*-methylcytidine (2CMC).

### 2.5. Compound **6** Does Not Directly Inhibit the Norovirus Polymerase

Given that compound **6** acts at the same time point in the viral replication cycle as polymerase inhibitors, we could assume that the compound inhibits the active site of the MNV polymerase by directly competing with their natural substrate, as a nucleoside analog would. However, no direct competition between the norovirus polymerase and the natural substrate was observed when the compound was present.

### 2.6. Selection of Compound **6**^res^ MNV Variants

**Figure 4:**
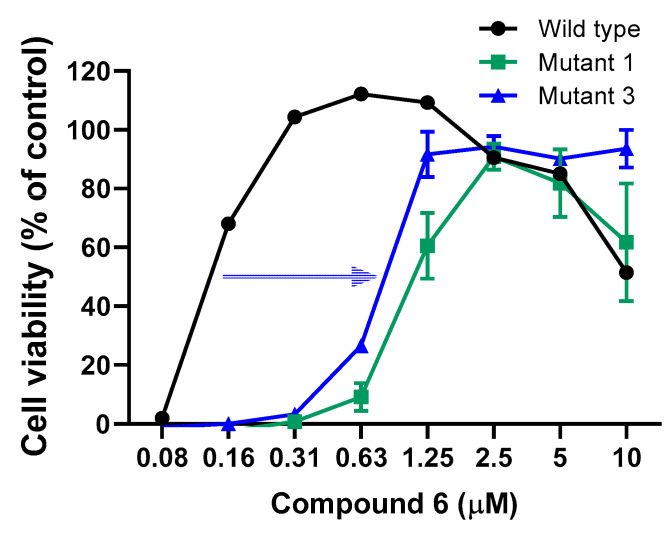
**Phenotypical confirmation of compound 6^res^ MNV in RAW 264.7 cells.** Confirmation of the compound **6**^res^ MNV variants, which were independently selected after 30 passages (6 months). Results are mean values (±SEM) of 3 independent experiments.

**Figure 5:**
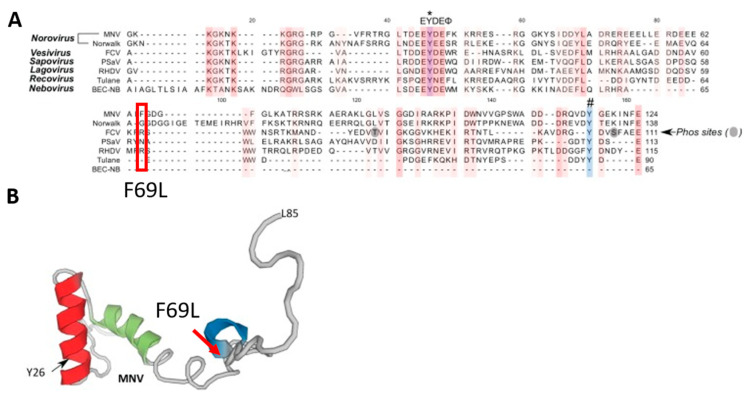
**The F69L mutation in the VPg protein detected in compound 6^res^ MNV variants**. One VPg (F69L) mutation detected in the genome of compound 6^res^ MNV variants. (**A**) Amino acid alignment of VPg sequences among representatives of calicivirus genera: MNV (DQ285629), Norwalk (AF093797), FCV (M86379), PSaV (AF182760), RHDV (Z49271), Tulane (EU391643) and BEC-NB (AY082891). Alignment of the first 20 nucleotides of the genomic. An asterisk (*) indicates the conserved tyrosine (Y) residue essential for calicivirus replication. A hash (#) indicates the Y residue identified necessary for MNV nucleotidylylation using an in vitro biochemical approach. (**B**) The solution structure of the MNV VPg protein (PDB: 2MG4). The MNV VPg structure encompasses amino acids G11 to L85. Reprinted from Ref. [5].

**Figure 6:**
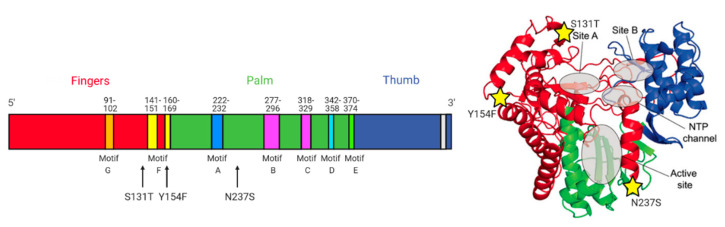
**Mutations detected in the RdRp of compound 6^res^ MNV variants.** Three mutations in the RdRp (S131T, Y154F, and N237S) in the genome of compound **6**^res^ MNV variants. Reprinted from ref. [9]. (Created with www.biorender.com, accessed on 10 September 2021).

To evaluate the dynamics of emerging resistance against compound **6** we cultured the MNV under sub-optimal compound concentrations. After six months, three independent resistant variants were selected after 30 passages. Of those, two of these variants (mutant 1 and 3 in Figure 4) resulted in a modest shift in EC_50_ values of 8.6-fold or 6.2-fold, thus attesting the very high barrier to resistance of this compound class (Figure 4). These two resistant variants were objects of whole genome sequencing by NGS to determine the potential mutations linked to the resistant phenotype. Upon deep sequencing, the same four mutations were detected in both mutant 1 and 3, one mutation in the VPg (F69L) and three mutations in the RdRp (S131T, Y154F, and N237S) (Figure 5 and Figure 6). Within the VPg protein, there are only a limited number of amino acids which are highly conserved across all calicivirus genera, only a lysine rich N-terminal region and a central motif containing the EYDE sequence (Figure 5) [5]. The tyrosine within this motif, positions 24 and 26 of FCV and MNV respectively, is possibly the site for VPg nucleotidylylation and is an essential amino acid for viral infectivity [6,7,8]. The F69L mutation acquired in the compound **6**^res^ MNV is located right before a helix structure (Figure 5), this part of the VPg protein is highly mobile and could become ordered once in contact with other viral proteins; thus we cannot exclude an involvement of this region in terms of infectivity. As the amino acid change (F → L) results in a larger amino acid, it could imply that there is steric hindrance that prevents the compound from binding and leading to the resistant phenotype.

Within the RdRp there are highly conserved sequences and structural motifs A-H. The S131T and Y154F mutation are both positioned around motif F (Figure 6). The positively charged side chains of basic amino acids in motif F contribute electrostatic interactions to position the triphosphate moiety of the bound NTP [10]. The N237S mutation is located between motif A and B. Motif A contains two highly conserved Asp residues that participate in the ”two-metal ion” mechanism of catalysis found in most nucleic acid polymerases [11]. Motif B contains two highly conserved peptide segments that form a binding pocket to recognize the ribose moiety of the NTP [10]. Overall, the mutations in the RdRp are not clustered together or are not directly within a conserved motif or active site. We could therefore also consider that this molecule might act as an allosteric inhibitor of the RdRp, like suramine or NAF2 [12].

To understand which mutation(s) play a role in the compound **6**^res^ profile, we reverse-engineered these mutations into the MNV backbone and generated full-length infectious mutant MNV clones by transfection into Huh-7 Lunet cells expressing the CD300lf receptor. The S131T and Y154F mutant viruses were successfully recovered, while we were not yet able to recover the F96L and N237S mutant viruses. Introduction of the single mutations S131T or Y154F did not result in more resistant virus and thus no shift in EC_50_ of compound **6** was observed (Figure S1). As controls, we included 2CMC and rupintrivir. Both the S131T and Y154F mutant viruses remained equally sensitive to 2CMC and rupintrivir.

## 3. Discussion

In the search for an antiviral against HuNoV infections, we performed a large-scale antiviral drug screen, which included ~1000 drug-like small molecules from the Drug Design and Synthesis Centre (Sapienza University, Rome) for their potential anti-norovirus activity. We here report on the anti-norovirus activity of the 3-((3,5-dimethylphenyl)sulfonyl)-5-chloroindole-N-(phenylmethanol-4-yl)-2.carboxamide analog, compound **6**, againstmultiple norovirus genotypes.

The early hit compound 1 (3-((3,5-dimethylphenyl)sulfonyl)-5-chloroindole-N-(phenylmethanol-4-yl)-2.carboxamide), inhibited the replication of MNV and the HuNoV GI.1 replicon in vitro with EC_50_ values around 0.5 µM and 2 µM, respectively. The analog, compound 6, had an improved antiviral activity as it inhibited MNV and the HuNoV GI.1 replicon with EC_50_ values around 0.2 µM and 1.7 µM, respectively. In addition, compound 6 had a better antiviral effect against MNV and the HuNoV GI.1 replicon than 2CMC, which is currently used as the benchmark antiviral compound in norovirus research [13,14].

A first insight into the mechanism of action of compound **6** was achieved by studying the effect of adding this compound at different time points during infection. We determined that compound **6** acts at a post-entry step, most likely acting at the time viral genome replication starts. In fact, the presence of compound **6** is required at the same time points as that of the nucleoside analog 2CMC [14]. Hence, the first hypothesis was that compound **6** was also an RdRp targeting compound. However, a polymerase assay showed that the compound could not directly inhibit the active site of the enzyme. Still, compound **6** rendered the catalytic site of the MNV RdRp less efficient, thus it could act as an allosteric inhibitor of the RdRp.

Resistance development is a major obstacle in antiviral therapy, and almost all active antiviral agents have shown to select for resistance mutations. To assess whether resistant variants to compound **6** would easily arise (thus the class of compounds would have a low barrier to resistance) or if many passages (months) and multiple mutations would be required to confer resistance to the compound (high barrier to resistance), we passaged the virus in the presence of compound **6** up to 30 consecutive times [15,16,17,18,19]. The latter indicates a high barrier to resistance similar to 2CMC [14]. Out of the three mutants independently selected, two mutants (1 and 3) showed a reasonable shift in EC_50_ against compound **6** (6–8 fold). After deep sequencing, we found these two mutants had the same exact four mutations, one mutation in the VPg (F69L) and three mutations in the RdRp (S131T, Y154F, and N237S). By reverse engineering S131T and Y154F as single mutations into the MNV backbone, we did not find a markedly compound **6**^res^ phenotype but the S131T did confer a small 2-fold shift in EC_50_ value. Insertion of the other single mutations or combination of mutations is ongoing. Since there is a possibility that the mutant viruses would replicate without inducing a cytopathic effect, which is the used indicator of viral replication, we also measured the levels of viral RNA in samples by RT-qPCR after three passages in BV-2 cells. Despite repeated passage, we found no signs of viral RNA replication. This might also suggest that these amino acid changes make the virus unviable and that these amino acids play a critical role in the viral replication.

We can speculate that the mutations acquired in the RdRp play an important role in the resistance against compound **6**. However, in the case of noroviruses there are two equally active versions of its polymerase (Pol), the mature polymerase and a protease-polymerase precursor protein (ProPol), the latter thus contains both protease and polymerase activities [7]. The nucleotidylylation of VPg can be done by ProPol more efficiently than by polymerase alone [20]. It could therefore be possible that the acquired mutations play a role in this VPg ProPol interaction or cause a different folding of the PropPol structure and therefore prevent this uridylation step and thereby inhibit the viral RNA replication. This hypothesis is hard to confirm, as there is no crystal structure of the VPg ProPol structure available. However, the interaction site of RdRp and VPg is known for picornaviruses, of which the RdRp is highly similar to that of noroviruses [21]. To understand if these compounds might have a protein-protein interaction-based mechanism, the chemical scaffold was modified on the specific groups that are expected to hamper such interactions. However, with these analogs there was no difference in inhibition observed.

The high barrier to resistance is a crucial property of an antiviral drug, preferably multiple rather than single mutations are required, and resistant variants have a seriously compromised fitness. As selection for resistant virus showed to be so difficult, we need to take into consideration that this compound might impede an interaction with a cellular factor. As viruses require cellular factors to translate their transcripts, targeting these offers another strategy to develop broad antiviral drugs.

## 4. Materials and Methods

### 4.1. Viruses and Cells

Murine norovirus (MNV, strain MNV-1.CW1) was propagated as described earlier [22]. HG23 cells (Norwalk virus replicon-bearing Huh-7 cells kindly provided by Kyeong-Ok Chang, Kansas State University) and RAW 264.7 cells were maintained as described earlier [13,23]. Cells were incubated at 37 °C in a humidified atmosphere of 5% CO_2_. To obtain each virus stock, once full CPE was observed, cells underwent two freeze-thaw cycles and the virus was harvested from the supernatants after centrifugation (10 min, 1000× *g*) and stored at −80 °C. The viral titer was determined by endpoint titration.

### 4.2. Compounds

The compound library was synthetized and supplied by Prof. Romano Silvestri (Sapienza University, Rome, Italy) and dissolved in dimethyl sulfoxide (DMSO, VWR Chemicals, Leuven, Belgium).

### 4.3. Antiviral and Cytotoxicity Assay

Norovirus: The experiment was performed as described earlier [13,23]. In short, RAW 264.7 cells (1 × 10^4^ cells/well) were infected with MNV.CW1 in the presence of a dilution series of compounds. Antiviral activity and cytotoxicity were determined using a colometric assay using 3-(4,5-dimethylthiazol-2-yl)-5-(3-carboxymethoxyphenyl)-2-(4-sulfophenyl)-2H-tetrazolium (MTS). The 50% effective concentration (EC_50_) was defined as the compound concentration that protected 50% of the cells from CPE. The cell viability % was calculated as (OD_treated_/OD_CC_) × 10 and the 50% cytotoxic concentration (CC_50_) was defined as the compound concentration that reduces the number of viable cells by 50%.

HuNoV GI.1 replicon: The experiment was performed as described earlier with minor modifications [13]. In short, HG23 cells (7.5 × 10^2^ cells/well) were seeded into the wells of a 96-well plate without G418 (Geneticin Selective Antibiotic). After 24 h of incubation, serial dilutions of compounds were added. Cells were further incubated for 72 h, then cells were collected for RNA load quantification by RT-qPCR. To determine relative levels of Norwalk virus replicon RNA, β-actin was used as a normalizer and ratios were calculated as previously described [13]. The EC_50_ values were defined as the compound concentration that resulted in 50% reduction of the relative HuNoV GI.1 replicon RNA levels.

### 4.4. Virus Yield Assay and RT-qPCR

The supernatants of treated or untreated infected cells were harvested. The viral RNA was isolated from supernatant using the NucleoSpin RNA Virus Kit (Macherey–Nagel), according to the manufacturer’s protocol and was quantified by RT-qPCR. A one-step RT-qPCR was performed in a reaction mixture of 20 μL containing 10 μL reaction mix from the iTaq Universal Probes One-Step Kit (Bio-Rad), 0.5 μL reverse transcriptase, 4 μL template RNA, primers, and probe [24].

### 4.5. Time-of-Drug-Addition Assay (TOA)

RAW 264.7 cells (4 × 10^5^ cells/mL) were infected with MNV (MOI of 1). After 1 h at 4 °C, cells were washed with cold (4 °C) DMEM to remove unbound viruses. Cells were incubated at 37 °C to start the infection. To study the replication kinetics of MNV supernatant and cells were harvested, from untreated cultures, every 2 h until 24 h post-infection (pi) and viral RNA was quantified by RT-qPCR. In parallel, another set of infected cultures were treated with compound. The compound was added at 2 h intervals and cultures were further incubated until 24 h pi, supernatant and cells were collected separately for determination of the viral RNA by RT-qPCR. The isolation of the extracellular MNV RNA from cell culture supernatant was done by the NucleoSpin RNA Virus Kit (Macherey–Nagel), and the intracellular RNA was extracted from cells by the RNeasy kit (Qiagen).

### 4.6. In Vitro Enzymatic Inhibition Assay with MNV RdRp

Compound **6** was tested to determine its effects on the polymerase activity. The formation of dsRNA, from a single-stranded poly(U) template (Sigma-Aldrich, 10 µg) and 100 µM ATP, was followed for 1 h at 30 °C on a TECAN Infinite 200 PRO microplate reader, measuring the growth of PicoGreen fluorescence (Ex/Em = 485/530 nm) after the addition of 3.4 µM MNV RdRp and of increasing amounts of compound **6** (from 0 to 100 µM), in a reaction mixture containing 20 mM Tris/HCl (pH 7.5), 25 mM NaCl, 0,3 mM MnCl_2_, 5 mM MgCl_2_, 1 mM DTT, 2 U RiboLock Ribonuclease inhibitor (Life technologies). The final fluorescence values were calculated as the average of four independent experiments. Measurement of the activity (i.e., linear slope of fluorescence increase in time) vs. compound **6** concentration was used to estimate the IC_50_ value of the compound with the program GraFit5 (Erithacus software).

### 4.7. Resistance Selection—MNV

RAW 264.7 cells (1 × 10^4^ cells/well) were seeded into the wells of 96-well plate and were infected with MNV.CW1 in the presence of a dilution series of compounds. When CPE was visible in the virus control (no compound), all the wells were scored for visible CPE. Virus was harvested from the wells in which CPE was observed under the highest compound pressure. The resistant virus was then diluted to 1:5 and used to infect a new 96-well plate under the same conditions to continue. After 25 passages (~6 months), the viral RNA was isolated using the NucleoSpin RNA Virus Kit (Macherey–Nagel). MNV^res^ viruses were analyzed by deep sequencing by the group of Prof. Matthijnssens as described previously [25]. The assembled sequence of the untreated MNV, that was passaged in parallel, was used as reference to align the MNV variants that were under compound pressure [26].

### 4.8. Site Directed Mutagenesis

The desired mutations were introduced into the MNV.CW3 cDNA clones [27] using the QuikChange II XL mutagenesis kit (Agilent) according to the manufacturer’s instructions. The presence of the desired mutation in the produced construct was confirmed by PCR and sequencing was done by Macrogen Inc. Sequences were analyzed and mutations were detected using the Geneious software.

### 4.9. Growing of Resistant Virus Stock

The MNV.CW3 mutant viral stocks were obtained by transfecting the mutated plasmid pMNV CW3 (kindly provided by Prof. Wobus, University of Michigan) in Huh 7 Lunet cells expressing the MNV receptor CD300lf (kindly provided by Prof. Taube, University of Lübeck) [28]. Huh-7 CD300lf cells were cultured under selective pressure of G418. The Huh-7 CD300lf cells were seeded in 6-well plates at a density of 2 × 10^5^ cells/mL in 2.5 mL of complete media. After 24 h, the cells were transfected with 2.5 μg of pMNV CW3 plasmid DNA using the TransIT mRNA Transfection Kit (Mirus Bio Corporation) according to the manufacturer’s protocol. Red fluorescent protein (RFP) was used as a positive control for transfection. Transfected cells were incubated for 48 h, cells underwent two freeze thaw cycles and the virus was harvested from the supernatants after centrifugation (10 min, 1000× *g*) and stored at −80 °C. Virus stocks were passaged for three times on BV-2 cells and followed by two consecutive passages on RAW 264.7 cells. Viral titers were determined by serial dilutions on RAW 264.7 cells seeded in 96-well plates.

### 4.10. Antiviral Assay with Mutant Viruses

The assessment of the antiviral activity of compounds on resistant viruses was performed similarly as the regular antiviral assay on MNV. First, the titer of the resistant viruses was determined by endpoint titration. A concentration of 30× TCID_50_ was used for each virus in the antiviral assays. RAW 264.7 cells (1 × 10^4^ cells/well) were seeded in the presence of a dilution series of compounds and infected. Antiviral activity was determined using the MTS method. The 50% effective concentration (EC_50_) was defined as the compound concentration that protected 50% of the cells from CPE.

### 4.11. Chemistry

All reagents and solvents were handled according to the material safety data sheet of the supplier and were used as purchased without further purification. Organic solutions were dried over anhydrous sodium sulfate. Evaporation of solvents was carried out on a Büchi Rotavapor R-210 equipped with a Büchi V-850 vacuum controller and a Büchi V-700 vacuum pump. Column chromatography was performed on columns packed with silica gel from Merck (70–230 mesh). Silica gel thin layer chromatography (TLC) cards from Merck (silica gel precoated aluminum cards with fluorescent indicator visualizable at 254 nm) were used for TLC. Developed plates were visualized with a Spectroline ENF 260C/FE UV apparatus. Melting points (mp) were determined on a Stuart Scientific SMP1 apparatus and are uncorrected. Infrared (IR) spectra were recorded on a PerkinElmer Spectrum 100 FT-IR spectrophotometer equipped with a universal attenuated total reflectance accessory. IR data were acquired and processed by PerkinElmer Spectrum 10.03.00.0069 software. Band position and absorption ranges are given in cm^−1^. Proton nuclear magnetic resonance (1H NMR) spectra were recorded with a Varian Mercury (300 MHz) or a Bruker Avance (400 MHz) spectrometer in the indicated solvent, and the corresponding fid files were processed by MestreLab Research SL MestreReNova 6.2.1-769 software. Chemical shifts are expressed in δ units (ppm) from tetramethylsilane. The purity of tested compounds was checked by high pressure liquid chromatography (HPLC). The purity of the tested compounds was found to be >95%. The Thermo Fisher Scientific Inc. Dionex UltiMate 3000 HPLC system consisted of an SR-3000 solvent rack, an LPG-3400SD quaternary analytical pump, a TCC-3000SD column compartment, a DAD-3000 diode array detector, and an analytical manual injection valve with a 20 μL loop. Samples were dissolved in acetonitrile (1 mg/mL). HPLC analysis was performed by using a Thermo Fisher Scientific Inc. Acclaim 120 C18 column (5 µm, 4.6 mm × 250 mm), at 25 ± 1 °C with an appropriate solvent gradient (acetonitrile/water), flow rate of 1.0 mL/min and signal detector at 206, 230, 254, and 365 nm. Chromatographic data were acquired and processed by Thermo Fisher Scientific Inc. Chromeleon 6.80 SR15 Build 4656 software.

#### 4.11.1. General Procedure A

To a solution of the protect derivative (0.11 mmol) in tetrahydrofuran (20 mL/mmol) tetrabutylammonium fluoride (solution 1 M in THF, 0.17 mmol) was added and the resulting mixture was stirred at 25 °C for 3 h. The solvent was removed from the reaction mixture and the resulting product was purified by column chromatography (silica gel, chloroform:ethanol = 9:1).

#### 4.11.2. General Procedure B

A mixture of acid (0.30 mmol), triethylamine (0.90 mmol), amine (0.90 mmol), and (benzotriazol-1-yloxy)tripyrrolidinophosphonium hexafluorophosphate (0.30 mmol) in *N*,*N*-dimethylformamide (20 mL/mmol) was stirred at 40 °C for 18 h under an argon stream. After cooling, the reaction mixture was diluted with water and extracted with ethyl acetate. The organic layer was washed with brine, dried, and filtered. Evaporation of the solvent gave a residue that was purified by column chromatography (silica gel, *n*-hexane:ethyl acetate = 1:1).

#### 4.11.3. General Procedure C

To a solution of 4-(hydroxymethyl)aniline (0.89 mmol) in chloroform (10 mL/mmol) *tert*-butyldimethylsilyl chloride (0.89 mmol) and imidazole (0.97 mmol) was added. The reaction mixture was stirred at 25 °C for 1 h, then diluted with water and extracted with ethyl acetate. The organic layer was washed with brine, dried and filtered. Evaporation of the solvent gave a residue that was purified by column chromatography (silica gel, *n*-hexane:ethyl acetate = 2:1).

5-Chloro-3-((3,5-dimethylphenyl)sulfonyl)-*N*-(4-(hydroxymethyl)phenyl)-1*H*-indole-2-carboxamide (compound **1**). It was synthesized according to general procedure A, starting from 19. Yield 40%, mp 220–221 °C (from ethanol) as a white powder. ^1^H NMR (DMSO-*d_6_*, 300 MHz): δ δ 2.29 (s, 6H), 4.49 (d, *J* = 4.2 Hz, 2H), 5.18 (d, *J* = 4.3 Hz, 1H), 7.25–7.26 (m, 1H), 7.34-7.36 (m, 3H), 7.55 (d, *J* = 6.5 Hz, 1H), 7.64 (s, 2H), 7.68 (d, *J* = 6.2 Hz, 2H), 7.92–7.93 (m, 1H), 10.85 (br s, disappeared after treatment with deuterium oxide, 1H), 13.21 ppm (br s, disappeared after treatment with deuterium oxide, 1H). IR: ν 1632 and 2984 cm^−1^.

*N*-Benzyl-5-chloro-3-((3,5-dimethylphenyl)sulfonyl)-1*H*-indole-2-carboxamide (compound **2**). It was synthesized as previously reported [3].

5-Chloro-3-((3,5-dimethylphenyl)sulfonyl)-*N*-(2-hydroxyethyl)-1*H*-indole-2-carboxamide (compound **3**). It was synthesized as previously reported [4].

5-Chloro-3-((3,5-dimethylphenyl)sulfonyl)-*N*-(4-hydroxybenzyl)-1*H*-indole-2-carboxamide (compound **4**). It was synthesized according to general procedure B, starting from compound **18** and 4-hydroxybenzylamine. Yield 60%, mp 236–237 °C (from ethanol), as a cream colour powder. ^1^H NMR (DMSO-*d_6_*, 300 MHz): δ 2.26 (s, 6H), 4.46 (d, *J* = 4.3 Hz, 2H), 6.73-6.75 (m, 2H), 7.22-7.24 (m, 3H), 7.33 (dd, *J* = 1.6 and 6.6 Hz, 1H), 7.51–7.53 (m, 1H), 7.55-7.56 (m, 2H), 7.94 (dd, *J* = 0.5 and 1.6 Hz, 1H), 9.32 (br s, disappeared after treatment with deuterium oxide, 1H), 9.34 (br s, disappeared after treatment with deuterium oxide, 1H), 13.03 ppm (br s, disappeared after treatment with deuterium oxide, 1H). IR: ν 1684 and 3105 cm^−1^.

5-Chloro-*N*-(4-(hydroxymethyl)phenyl)-3-(phenylsulfonyl)-1*H*-indole-2-carboxamide (compound **5**). It was synthesized according to general procedure A, starting from compound **20**. Yield 12%, mp 152–155 °C (from ethanol), as a pale white powder. ^1^H NMR (DMSO-*d_6_*, 300 MHz): δ 4.47 (d, *J* = 5.7 Hz, 2H), 5.17 (br t, *J* = 5.7 Hz, disappeared after treatment with deuterium oxide, 1H), 7.30–7.34 (m, 3H), 7.52–7.60 (m, 4H), 7.67–7.70 (m, 2H), 7.92–7.93 (m, 1H), 8.05-8.08 (m, 2H), 10.87 (br s, disappeared after treatment with deuterium oxide, 1H), 13.23 ppm (br s, disappeared after treatment with deuterium oxide, 1H). IR: ν 1523 and 3291 cm^−1^.

5-Chloro-3-((3,5-dimethylphenyl)sulfonyl)-*N*-(4-(hydroxymethyl)benzyl)-1*H*-indole-2-carboxamide (compound **6**). It was synthesized according to general procedure B, starting from compound **18** and (4-(aminomethyl)phenyl)methanol. Yield 18%, mp 124–126 °C (from ethanol), as a white powder. ^1^H NMR (DMSO-*d_6_*, 400 MHz): δ 2.28 (s, 6H), 4.50 (d, *J* = 5.6 Hz, 2H), 4.57 (d, *J* = 5.8 Hz, 2H), 5.17 (br t, *J* = 5.7 Hz, disappeared after treatment with deuterium oxide, 1H), 7.25–7.26 (m, 1H), 7.31 (d, *J* = 8.1 Hz, 2H), 7.32–7.35 (m, 1H), 7.40 (d, *J* = 7.9 Hz, 2H), 7.54 (d, *J* = 8.8 Hz, 1H), 7.59–7.60 (m, 2H), 7.94 (d, *J* = 1.8 Hz, 1H), 9.42 (br t, *J* = 5.4 Hz, disappeared after treatment with deuterium oxide, 1H), 13.06 ppm (br s, disappeared after treatment with deuterium oxide, 1H). IR: ν 1658 and 2984 cm^−1^.

5-Chloro-3-(3,5-dimethylbenzyl)-*N*-(4-(hydroxymethyl)benzyl)-1*H*-indole-2-carboxamide (compound **7**). It was synthesized according to general procedure B, starting from 16 and (4-(aminomethyl)phenyl)methanol. Yield 22%, mp 171–174 °C (from ethanol), as a white powder. ^1^H NMR (DMSO-*d_6_*, 400 MHz): δ 2.16 (s, 6H), 4.34 (s, 2H), 4.49 (t, *J* = 6.6 Hz, 4H), 5.16 (br t, *J* = 5.7 Hz, disappeared after treatment with deuterium oxide, 1H), 6.75–7.76 (m, 1H), 6.83 (s, 2H), 7.18 (dd, *J* = 2,0 and 8.7 Hz, 1H), 7.25–7.30 (m, 4H), 7.41 (d, *J* = 8.7 Hz, 1H), 7.55 (d, *J* = 1,2 Hz, 1H), 8.57 (br t, *J* = 5.6 Hz, disappeared after treatment with deuterium oxide, 1H), 11.54 ppm (br s, disappeared after treatment with deuterium oxide, 1H). IR: ν 1613 and 2917 cm^−1^.

5-Chloro-3-(3,5-dimethylbenzoyl)-*N*-(4-(hydroxymethyl)benzyl)-1*H*-indole-2-carboxamide (compound **8**). It was synthesized according to general procedure B, starting from compound **15** and (4-(aminomethyl)phenyl)methanol. Yield 15%, mp 271–274 °C (from ethanol), as a pale white powder. ^1^H NMR (DMSO-*d_6_*, 400 MHz): δ 2.30 (s, 6H), 4.24 (d, *J* = 5.6 Hz, 2H), 4.47 (d, *J* = 5.6 Hz, 2H), 5.14 (br t, *J* = 5.7 Hz, disappeared after treatment with deuterium oxide, 1H), 7.15 (d, *J* = 8.1 Hz, 2H), 7.24 (d, *J* = 8.2 Hz, 2H), 7.27–7.32 (m, 5H), 7.55 (d, *J* = 8.7 Hz, 1H), 9.56 (br t, *J* = 5.7 Hz, disappeared after treatment with deuterium oxide, 1H), 12.80 ppm (br s, disappeared after treatment with deuterium oxide, 1H). IR: ν 1646 and 2922 cm^−1^.

5-Chloro-3-((3,5-dimethylphenyl)thio)-*N*-(4-(hydroxymethyl)benzyl)-1*H*-indole-2-carboxamide (compound **9**). It was synthesized according to general procedure B, starting from compound **12** and (4-(aminomethyl)phenyl)methanol. Yield 15%, mp 212–215 °C (from ethanol), as a pale yellow powder. ^1^H NMR (DMSO-*d_6_*, 300 MHz): δ 2.11 (s, 6H), 4.42 (d, *J* = 5.0 Hz, 2H), 4.52 (d, *J* = 5.9 Hz, 2H), 5.13 (br t, *J* = 5.6 Hz, disappeared after treatment with deuterium oxide, 1H), 6.65-6.66 (m, 2H), 6.78-6.79 (m, 1H), 7.15–7.16 (m, 4H), 7.28 (dd, *J* = 2.0 and 8.8 Hz, 1H), 7.45 (d, *J* = 1.9 Hz, 1H), 7.53 (d, *J* = 8.6 Hz, 1H), 8.74 (br t, *J* = 5.6 Hz, disappeared after treatment with deuterium oxide, 1H), 12.51 ppm (br s, disappeared after treatment with deuterium oxide, 1H). IR: ν 1636 and 3219 cm^−1^.

5-Chloro-*N*-(4-(hydroxymethyl)benzyl)-3-(phenylsulfonyl)-1*H*-indole-2-carboxamide (compound **10**). It was synthesized according to general procedure B, starting from compound **13** and (4-(aminomethyl)phenyl)methanol. Yield 24%, mp 182–185 °C (from ethanol), as a white powder. ^1^H NMR (DMSO-*d_6_*, 300 MHz): δ 4.47 (d, *J* = 4.9 Hz, 2H), 4.53 (d, *J* = 5.1 Hz, 2H), 5.17 (br t, *J* = 5.2 Hz, disappeared after treatment with deuterium oxide, 1H), 7.27–7.38 (m, 5H), 7.49, 7.59 (m, 4H), 7.91–7.98 (m, 3H), 9.41 (br t, *J* = 4.8 Hz, disappeared after treatment with deuterium oxide, 1H), 13.07 ppm (br s, disappeared after treatment with deuterium oxide, 1H). IR: ν 1523 and 3214 cm^−1^.

5-Chloro-3-((3,5-dimethylphenyl)sulfonyl)-*N*-(4-hydroxyphenethyl)-1*H*-indole-2-carboxamide (compound **11**). It was synthesized according to general procedure B, starting from compound **14** and 4-hydroxyphenethylamine. Yield 56%, mp 231–234 °C (from ethanol), as a white powder. ^1^H NMR (DMSO-*d_6_*, 300 MHz): δ 2.29 (s, 6H), 2.76 (t, *J* = 7.7 Hz, 2H), 3.49 (q, *J* = 7.1 Hz, 2H), 6.67 (d, *J* = 8.2 Hz, 2H), 7.08 (d, *J* = 8.2 Hz, 2H), 7.24–7.31 (m, 2H), 7.50 (d, *J* = 8.8 Hz, 1H), 7.58–7.59 (m, 2H), 7.90–7.91 (m, 1H), 8.99 (br s, disappeared after treatment with deuterium oxide, 1H), 9.19 (br s, disappeared after treatment with deuterium oxide, 1H), 13.01 ppm (br s, disappeared after treatment with deuterium oxide, 1H). IR: ν 1620 and 2934 cm^−1^.

*N*-(4-(((*Tert*-butyldimethylsilyl)oxy)methyl)phenyl)-5-chloro-3-((3,5-dimethylphenyl)sulfonyl)-1*H*-indole-2-carboxamide (compound **19**). It was synthesized according to general procedure B, starting from compound **18** and 4-(((*tert*-butyldimethylsilyl)oxy)methyl)aniline. Yield 13% as an oil. ^1^H NMR (DMSO-*d_6_*, 300 MHz): δ 0.08 (s, 6H), 0.90 (s, 9H), 2.28 (s, 6H) 4.70 (s, 2H), 7.24–7.25 (m, 1H), 7.33–7.35 (m, 3H), 7.55 (d, *J* = 6.6 Hz, 1H), 7.63–7.64 (m, 2H), 7.69 (d, *J* = 6.3 Hz, 2H), 7.92 (d, *J* = 6.8 Hz, 1H), 10.85 (br s, disappeared after treatment with deuterium oxide, 1H), 13.25 ppm (br s, disappeared after treatment with deuterium oxide, 1H). IR: ν 1648 and 2929 cm^−1^.

4-(((*Tert*-butyldimethylsilyl)oxy)methyl)-*N*-((5-chloro-3-(phenylsulfonyl)-1*H*-indol-2-yl)methyl)aniline (compound **20**). It was synthesized according to general procedure B, starting from compound **17** and 4-(((*tert*-butyldimethylsilyl)oxy)methyl)aniline. The crude product was used without further purification.

4-(((*Tert*-butyldimethylsilyl)oxy)methyl)aniline (compound **21**). It was synthesized according to general procedure B, Yield 67% as an oil. ^1^H NMR (DMSO-*d_6_*, 300 MHz): δ 0.00 (s, 6H), 0.86 (s, 9H), 4.48 (s, 2H), 4.94 (br s, disappeared after treatment with deuterium oxide, 2H), 6.50 (d, *J* = 6.2 Hz, 2H), 6.93 ppm (d, *J* = 6.2 Hz, 2H). IR: ν 3529 and 4018 cm^−1^.

## 5. Conclusions

In conclusion, we here present the discovery, synthesis and activity of a novel class of anti-norovirus compounds. Overall, these findings confirm once again that targeting the HuNoV proteins is an attractive target for the development of norovirus antivirals. However, more research efforts are necessary in the search for effective HuNoV antiviral agents useful for the treatment of HuNoV infections.

## Figures and Tables

**Table 1:**
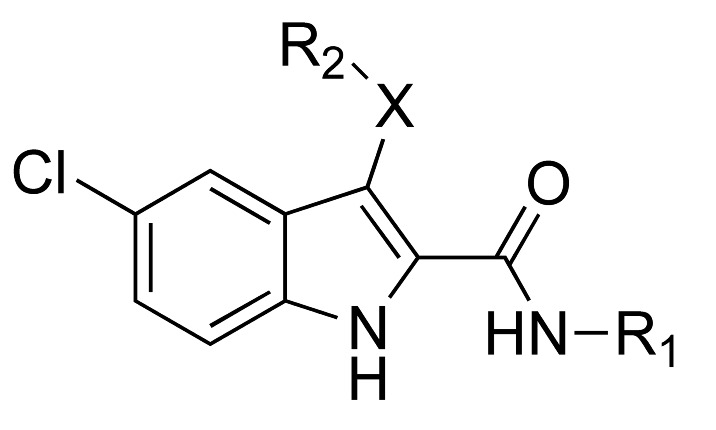
Anti-norovirus activity by compounds **1**–**11**.

				MNV ^a^	MNV	HuNoV Replicon ^b^
Compd	R_1_	R_2_	X	CC_50_ ± SD (µM) ^c,d^	EC_50_ ± SD (µM) ^c,e^	EC_50_ ± SD(µM) ^c,f^
1	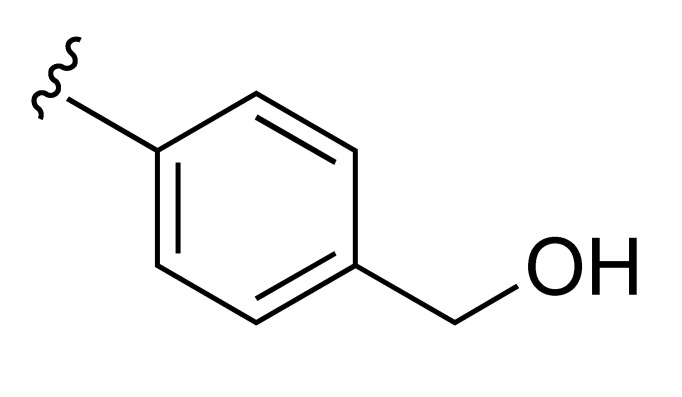	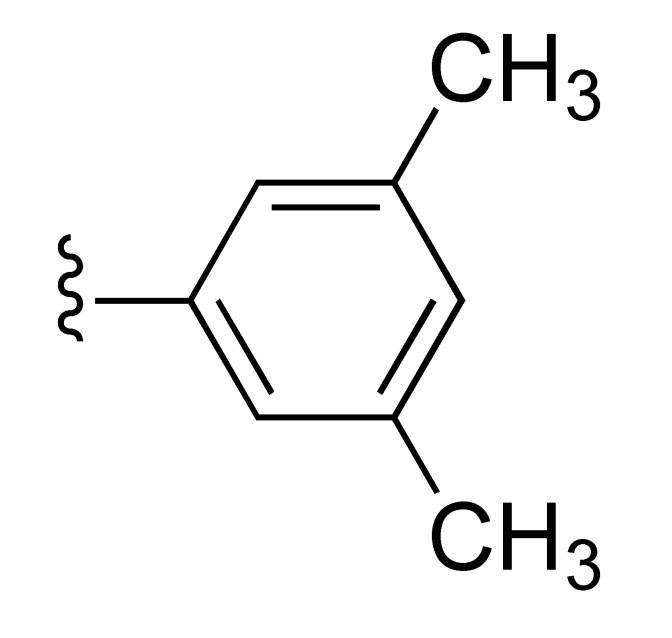	SO_2_	>100	0.52 ± 0.07	2 ± 1.2
2	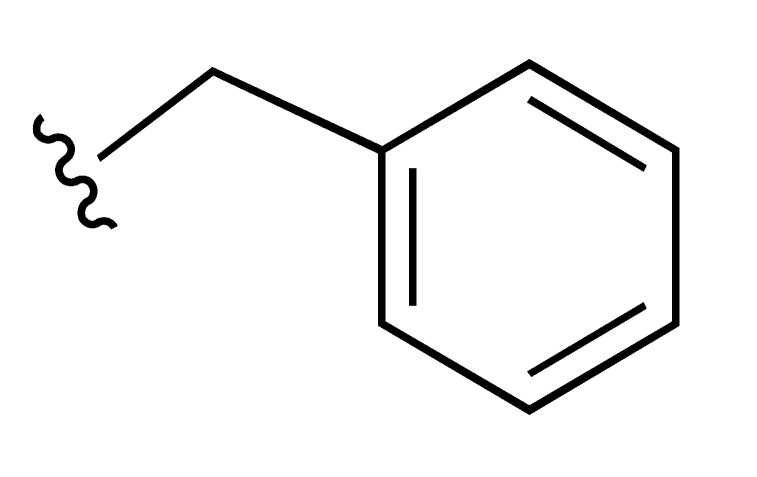	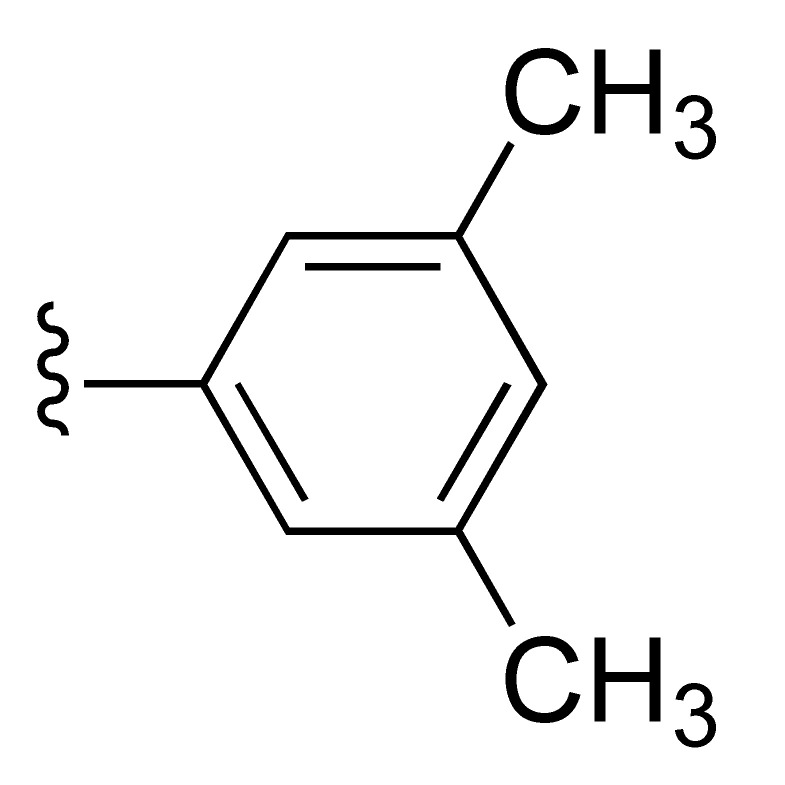	SO_2_	>100	>100	3.66 ± 0.66
3	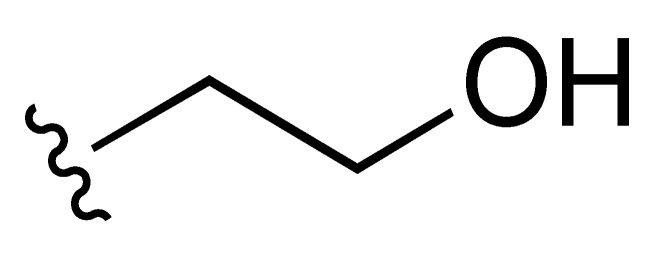	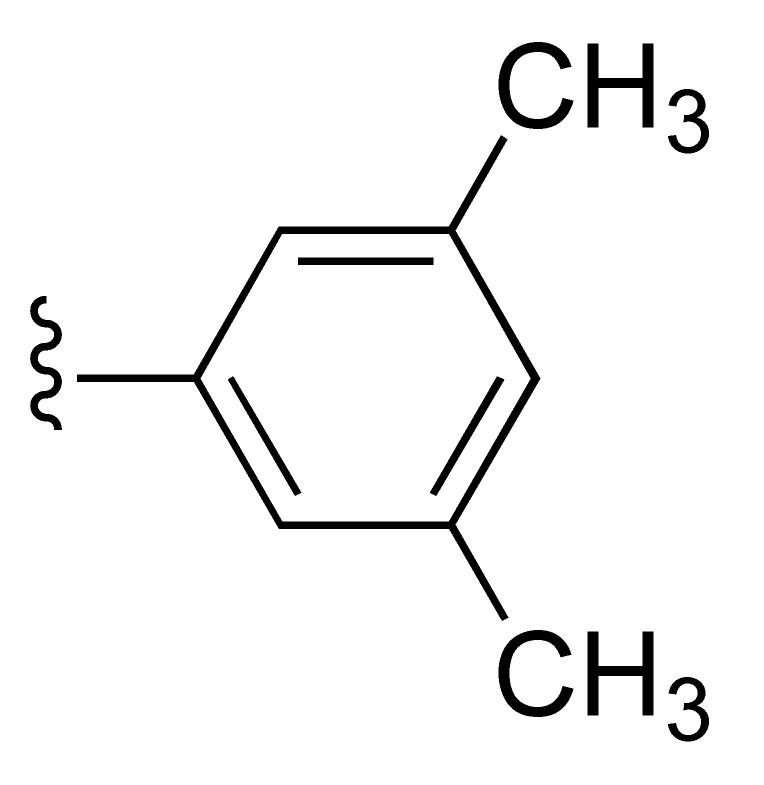	SO_2_	88.69 ± 4.75	>100	47.74 ± 3.39
4	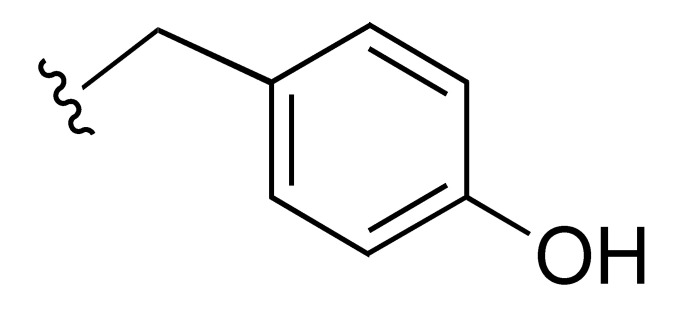	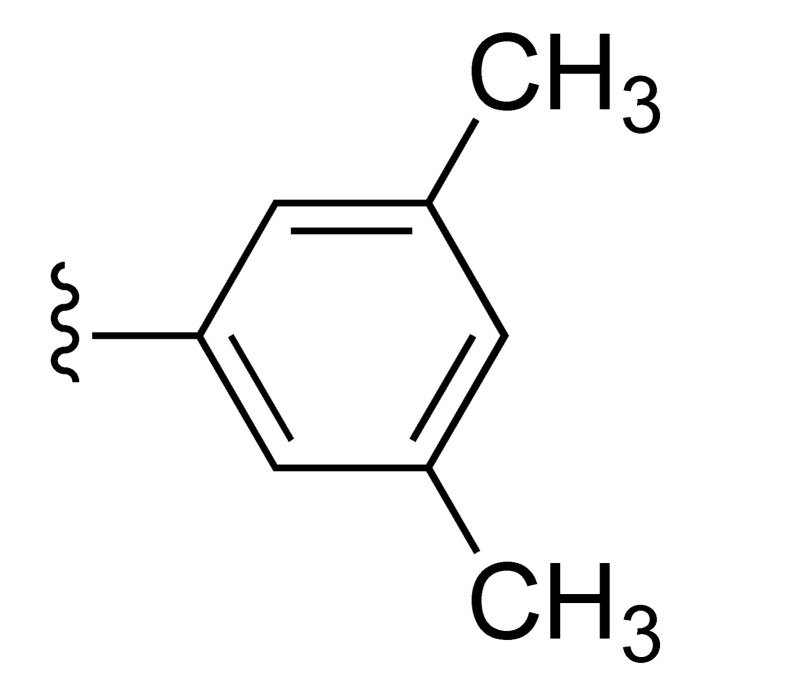	SO_2_	3.48 ± 1.10	>100	88.28 ± 4.75
5	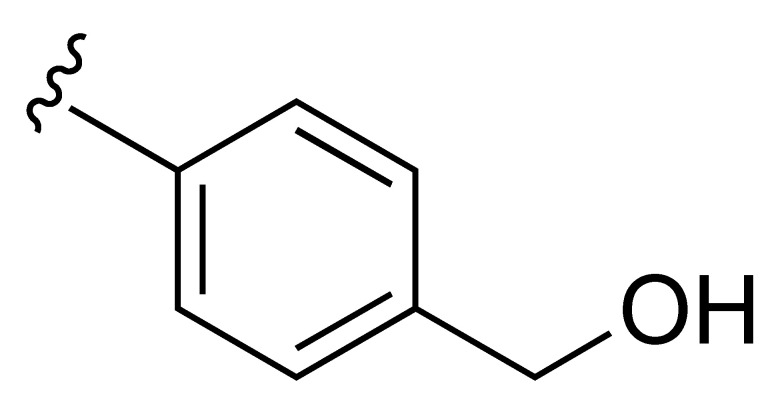	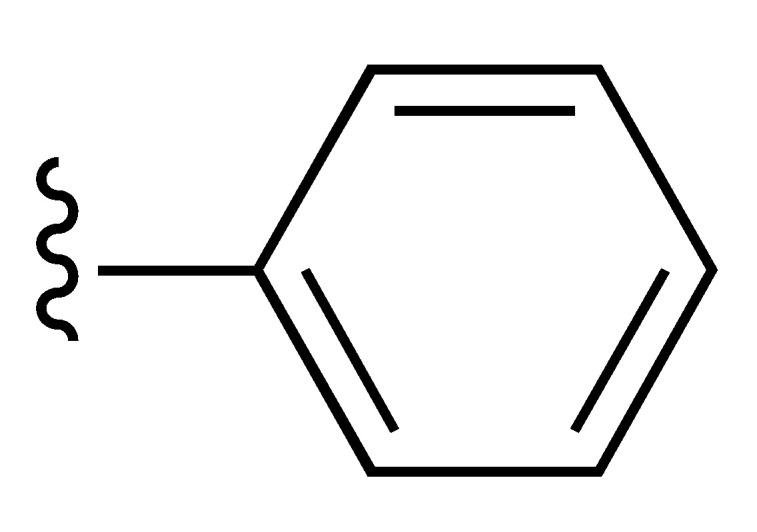	SO_2_	34.85 ± 5.03	>100	nd
6	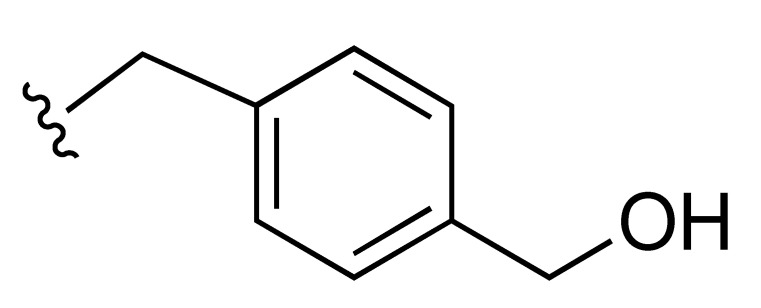	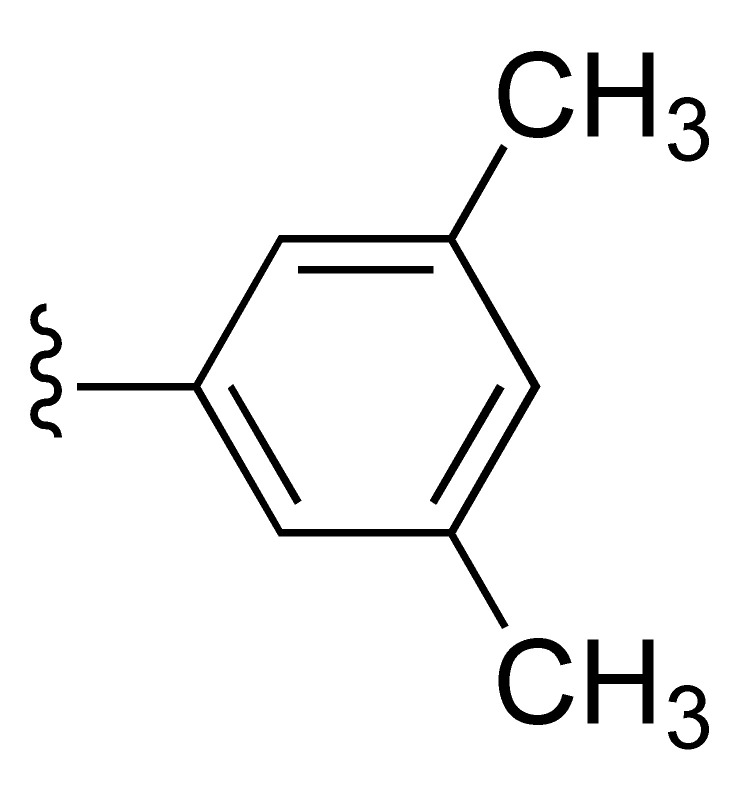	SO_2_	>12.50	0.16 ± 0.06	1.27 ± 0.76
7	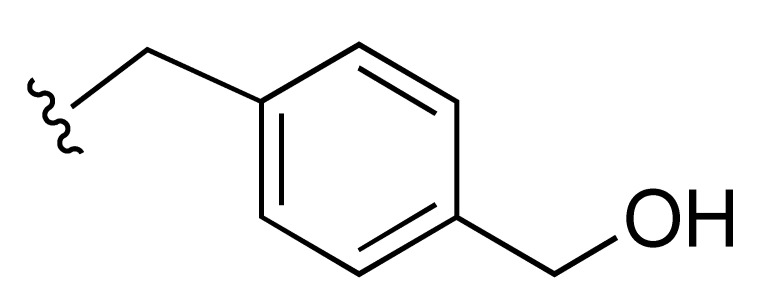	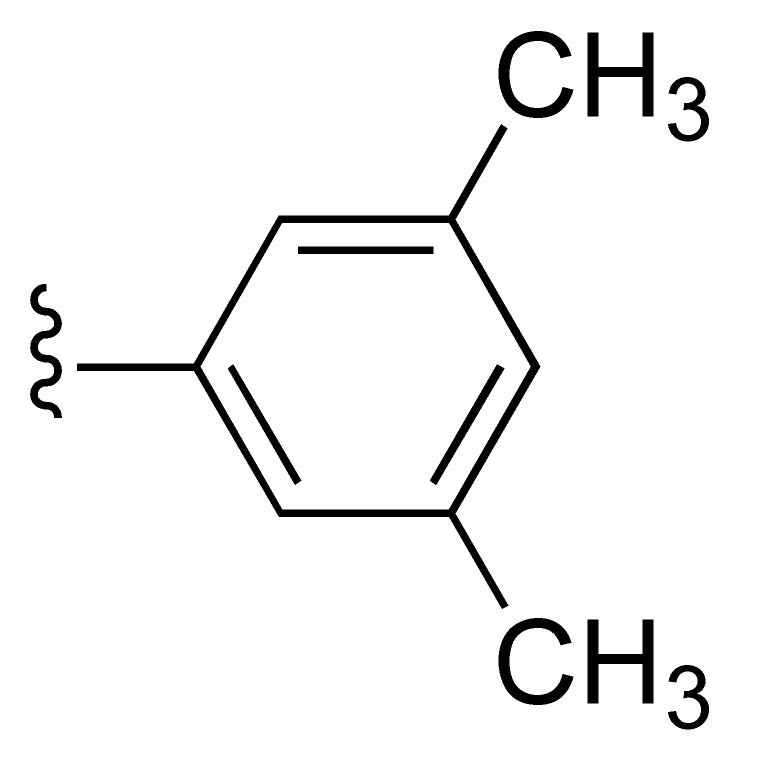	CH_2_	9.27 ± 6.60	0.84 ± 0.22	5.59 ± 2.47
8	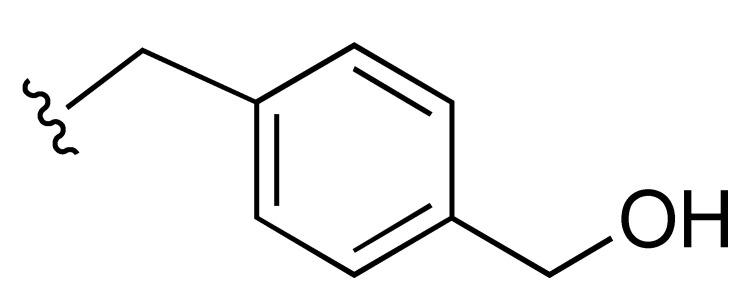	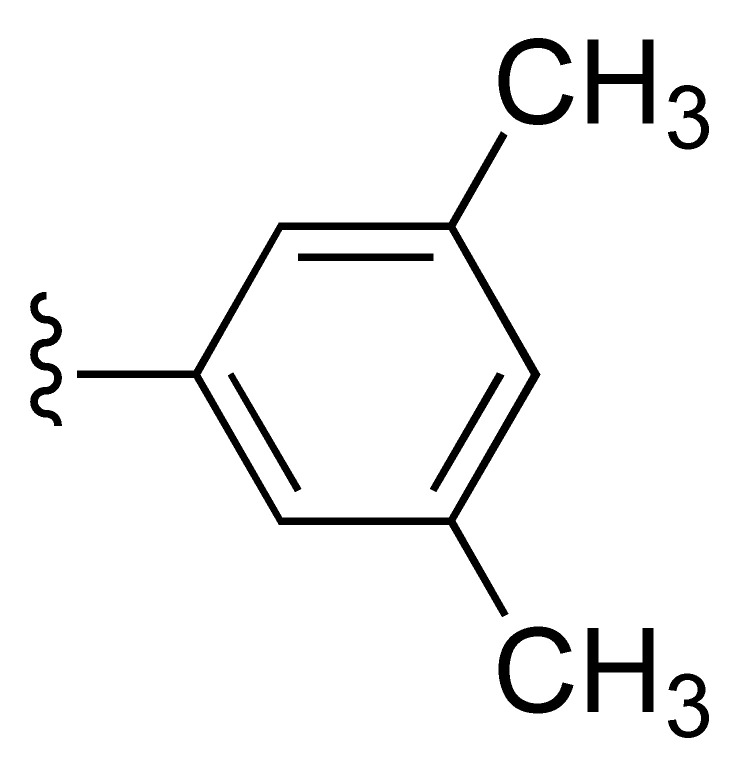	C=O	7.97 ± 15.29	>100	nd
9	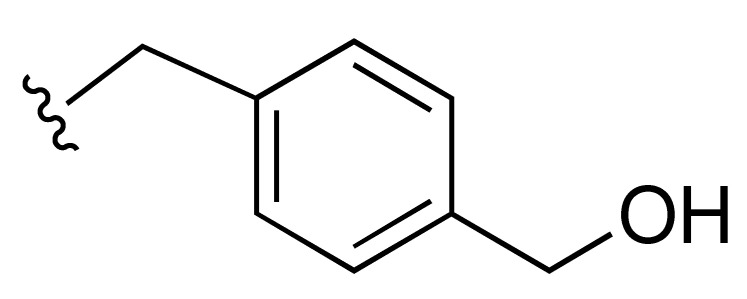	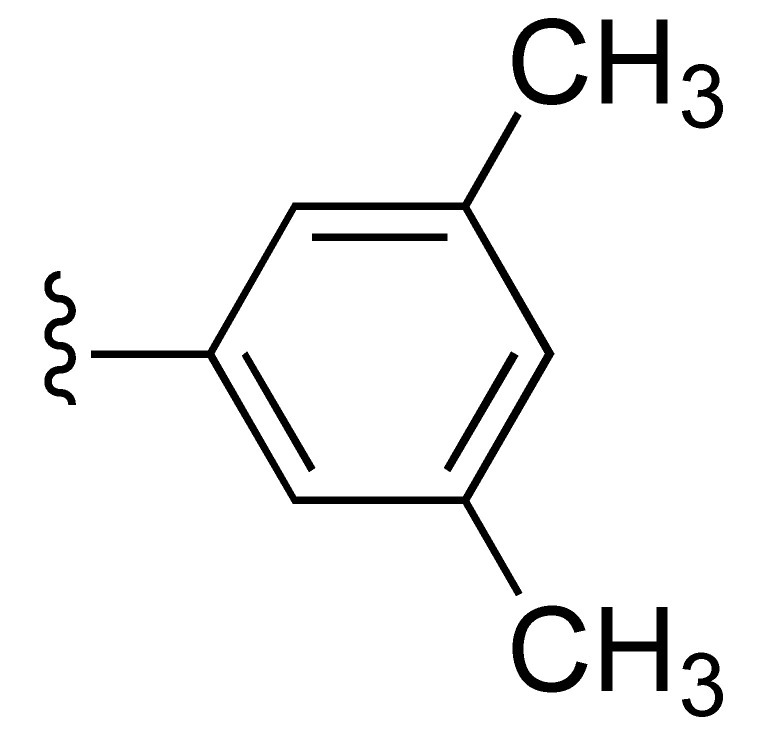	S	31.70 ± 8.01	4.39 ± 4.78	11.78 ±8.40
10	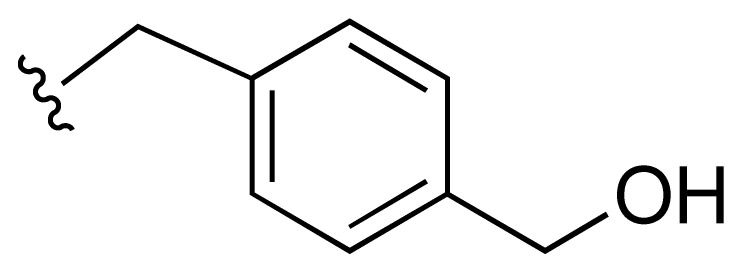	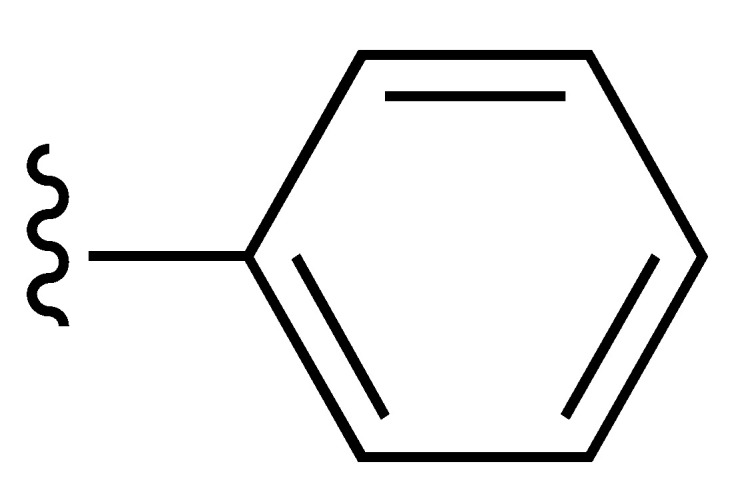	SO_2_	25.7 ± 34.94	0.63 ± 0.48	18.49 ± 16.27
11	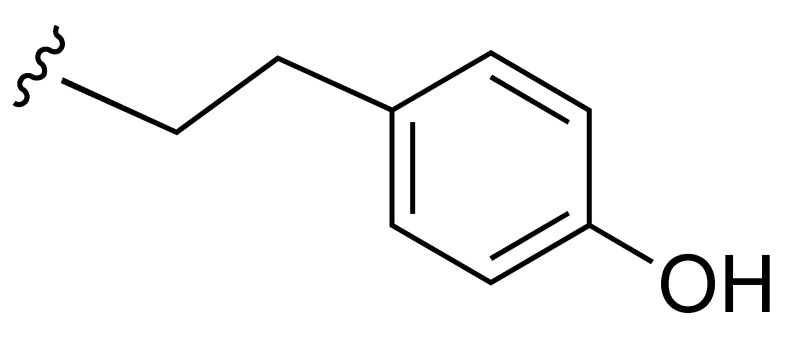	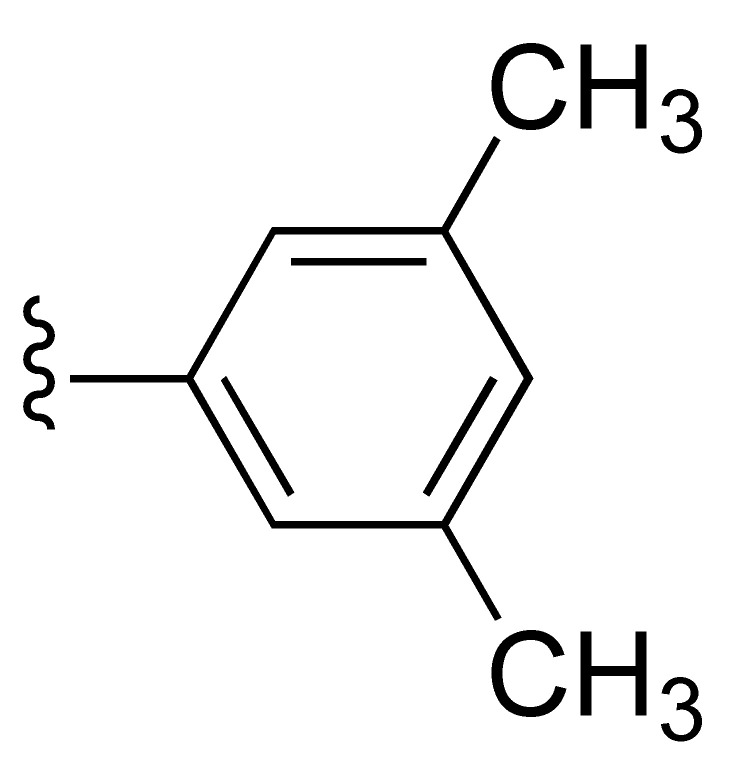	SO_2_	8.49 ± 8.09	35.95 ± 23.28	27.50 ± 28.69

^a^ MNV: Murine norovirus type 1, strain CW1; RAW cells, subtype 264.7. ^b^ Norwalk replicon (human GI.1). ^c^ Data are mean values of two to three independent experiments each one in triplicate. ^d^ CC_50_: cytotoxic concentration (µM) to induce 50% death of non-infected cells. ^e^ EC_50_: effective concentration (µM) to inhibit by 50% norovirus induced cell death. ^f^ EC_50_: effective concentration (µM) to inhibit by 50% of HuNoV replicon. nd: not determined.

## Data Availability

Data is contained within the article and Supplementary Materials.

## Supplementary Materials

The following are available online at https://www.mdpi.com/article/10.3390/ph14101006/s1, Figure S1: The antiviral effect of compound 6, 2CMC, rupintrivir against MNV, MNV/S131T and MNV/Y154F.
