# Supplementary material for: Discovery of a Novel Class of Norovirus Inhibitors with High Barrier of Resistance
Source: Pharmaceuticals (Basel). 2021 Sep 30;14(10):1006. doi: 10.3390/ph14101006 (PMC8537218; doi:10.3390/ph14101006)
Supplement: Supplementary file 1 [file pharmaceuticals-14-01006-s001.zip › pharmaceuticals-1371671-supplementary.pdf]

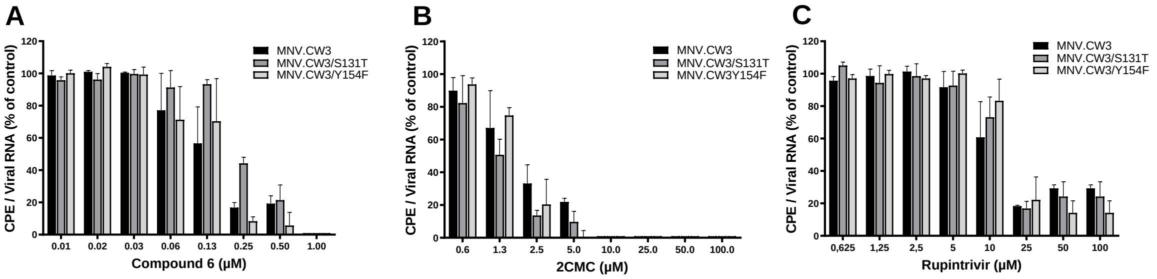

Supplementary Figure S1. The antiviral effect of compound 6, 2CMC, rupintrivir against MNV, MNV/S131T and MNV/Y154F. The antiviral activity of compound **6** (**A**), 2CMC (**B**) and rupintrivir (**C**) was quantified by means of virus-induced CPE reduction assay using a colometric read-out (black bars). Results are mean values (± SEM) of 3 independent experiments.
